# Nicotinamide *N*-methyltransferase is overexpressed in prostate cancer and correlates with prolonged progression-free and overall survival times

**DOI:** 10.3892/ol.2014.2287

**Published:** 2014-06-26

**Authors:** WEIMIN ZHOU, MING GUI, MIN ZHU, ZHI LONG, LIHUA HUANG, JUN ZHOU, LEYE HE, KUANGBIAO ZHONG

**Affiliations:** 1 Department of Urology, Third Xiangya Hospital, Central South University, Changsha, Hunan 410013, P.R. China; 2 Department of Nephrology, Third Xiangya Hospital, Central South University, Changsha, Hunan 410013, P.R. China; 3 Molecular Biology Research Center, Xiangya School of Medicine, Central South University, Changsha, Hunan 410078, P.R. China; 4 Center for Medical Experiments, Third Xiangya Hospital, Central South University, Changsha, Hunan 410013, P.R. China

**Keywords:** nicotinamide *N*-methyltransferase, prostate cancer, immunohistochemistry, prognosis

## Abstract

Nicotinamide ***N***-methyltransferase (NNMT) has been identified to be associated with tumorigenesis and the malignant transformation of numerous types of cancer. The aim of the present study was to explore the expression and prognostic significance of NNMT in prostate cancer (PCa). Immunohistochemical NNMT expression was examined in 26 cases of benign prostate hyperplasia (BPH), 18 cases of high-grade prostatic intraepithelial neoplasia (HGPIN) and 120 cases of PCa. While rarely expressed in BPH (8/26 cases; 30.8%), NNMT was found to be significantly upregulated in HGPIN (15/18 cases; 83.3%) and PCa (77/120 cases; 64.2%). Clinicopathological analysis revealed that NNMT expression was negatively correlated with Gleason score (P<0.001). Furthermore, Kaplan-Meier survival curves revealed that high NNMT expression was associated with prolonged progression-free survival (PFS) and overall survival (OS) times in patients with advanced PCa. Multivariate analysis showed that NNMT was an independent prognostic marker of PFS and OS in patients with advanced PCa. The results of the present study suggested that NNMT may contribute to the development of PCa and may potentially be a favorable prognostic marker for the survival of patients with advanced PCa.

## Introduction

Prostate cancer (PCa) is the second most commonly diagnosed form of cancer and the sixth leading cause of cancer-related mortality among males worldwide (1). For localized cancer, the five-year survival rate is ~100%, however, in patients exhibiting distant metastases, the five-year survival rate is 31% (2). For metastatic PCa, androgen deprivation therapy (ADT) remains the most important and primary treatment (3), which leads to symptomatic improvement and a reduction in the serum levels of prostate-specific antigen (PSA) in the majority of patients. However, the majority of these patients progress to hormone independence, the predominant obstacle that currently stops improvements in patient survival and quality of life (4,5). Therefore, it is important to determine markers that may identify patients expected to undergo disease progression, in order to optimize therapeutic decisions for individual patients. At present, the serum PSA level is the best prostate-specific tumor marker available and has also been demonstrated to be a useful prognostic indicator for survival in different clinical settings (6). However, in human PCa, serum PSA is not independently diagnostic or prognostic due to significant specificity and sensitivity limitations (7,8). Therefore, the identification of additional molecular markers is required to improve the screening criteria, diagnosis and prognosis of PCa.

Nicotinamide ***N***-methyltransferase (NNMT) is an *S*-adenosyl-L-methionine (SAM)-dependent cytoplasmic enzyme, which exhibits a critical role in the biotransformation and detoxification of numerous drugs and xenobiotic compounds (9,10). Previous studies have revealed the abnormal expression of NNMT in a number of cancers, and this may contribute to tumor development and progression, as well as resistance to radiotherapy and chemotherapy. In addition, NNMT has potential as a non-invasive biomarker of cancer in body fluids, including serum (11,12), saliva (13) and urine (14). To the best of our knowledge, the role of NNMT in PCa has not been identified thus far.

In the present study, immunohistochemistry was used to analyze the expression profile of NNMT in benign and malignant prostate specimens, as well as the correlation between NNMT expression and clinicopathological parameters. In addition, the prognostic significance of NNMT expression in advanced PCa was analyzed.

## Materials and methods

### Patient characteristics

This study was approved by the Research Ethics Committee of the Third Xiangya Hospital, Central South University (Changsha, China) and written informed consent was obtained from all patients. Primary PCa tissues were obtained by biopsy (84 cases) or radical prostatectomy (36 cases), high-grade prostatic intraepithelial neoplasia (HGPIN) tissues were obtained by biopsy (18 cases) and benign prostate hyperplasia (BPH) tissues were obtained from suprapubic prostatectomy (26 cases). All tissues were obtained between 2001 and 2005. The age ranges of the patients with PCa, HGPIN and BPH were 45–86 years (mean, 69.08±8.21), 59–84 years (mean, 71.50±6.55) and 55–85 years (mean, 70.00±8.16), respectively, and no significant differences were identified between the three groups. The pre-treatment PSA levels in the serum of the PCa, HGPIN and BPH patients were 0.16–216.0 ng/ml (mean, 48.46±40.92), 7.69–100.0 ng/ml (mean, 34.10±25.72) and 0.02–8.71 ng/ml (mean, 1.61±2.13), respectively. No patients with PCa received adjuvant hormonal therapy or chemotherapy prior to surgery.

The cancerous lesions were evaluated by two pathologists and consisted of tumors with a Gleason score of 5 (n=1), 6 (n=30), 7 (n =21), 8 (n=40), 9 (n=24), and 10 (n=4). The primary pathological tumor stages were pT1 in two cases, pT2a in four cases, pT2b in six cases, pT2c in 13 cases, pT3a in 15 cases, pT3b in 23 cases and pT4 in 57 cases. Furthermore, the regional lymph node stages were pN0 in 92 cases and pN1 in 28 cases, and the distant metastasis stages were pM0 in 64 cases and pM1 in 56 cases. Patients with advanced PCa at pT4 or (and) pN1 or (and) pM1 in this study were treated with maximal androgen blockade, and appropriate chemotherapy and radiotherapy were administered as required following the failure of hormone therapy.

### Patient follow-up

The survival rates of all the advanced PCa patients were analyzed, whereby the two end-points of progression-free survival (PFS) time and overall survival (OS) time were evaluated. The follow-up time ranged between seven and 96 months (median, 52 months). Disease progression during initial hormone therapy was defined by any of the following criteria: The identification of ≥1 novel bone metastases, attributable to metastatic disease; evidence of worsening of any existing bone metastases, attributable to metastatic disease; the identification of ≥1 novel extra-skeletal metastases or an increase in diameter of ≥20% (compared with the maximal diameter recorded prior to hormone administration) of any existing extra-skeletal metastases; and an increased serum PSA level (identified by three consecutive tests revealing increased serum PSA or a post-treatment PSA level of >4 ng/ml). Patients lost to follow-up and mortalities caused by diseases independent of PCa were regarded as censored data in the survival analysis.

### Immunohistochemistry

All samples were fixed with formalin and subsequently embedded in paraffin. Slices were cut at a 4-μm thickness and mounted on polylysine-coated glass slides. Sections were heated for 25 min at 56°C and then deparaffinized in xylene followed by rehydration using a graded series of ethanol concentrations. Immunohistochemistry was then performed following incubation for 20 min at 80°C with an antigen unmasking solution (Vector Laboratories, Burlingame, CA, USA). Endogenous peroxidase activity in the deparaffinized tissue was blocked by treatment with 3% hydrogen peroxide in phosphate-buffered solution (PBS) for 15 min at 25°C. Next, non-specific background staining was blocked by a 2-h incubation in 2% bovine serum albumin (Sigma-Aldrich, Oakville, ON, Canada) with 0.3% Triton X-100. The sections were then incubated with mouse monoclonal antibody against NNMT (1:150; Abcam, Cambridge, UK) overnight at 4°C. The sections were then rinsed in PBS with 0.1% Triton X-100 and incubated with biotinylated secondary antibody (Vector Laboratories) and streptavidin-horseradish peroxidase (Jackson ImmunoResearch, West Grove, PA, USA) for 1 h at 25°C. Following slide incubation for 2–5 min with 3,3′-diaminobenzidine (Vector Laboratories) to allow color development, all slides were counterstained with Mayer’s hematoxylin blue (Sigma-Aldrich) in 0.3% ammonia. For the positive controls of NNMT, normal liver tissues were used. For the negative controls, the sections were treated in an identical manner, with the exception of using primary antibody in order to confirm that these sections failed to develop specific staining.

### Immunohistochemical analysis

Immunostaining was scored by two independent pathologists who were blinded to the clinicopathological data and clinical outcomes of the patients. The scores of the two pathologists were compared and any discrepancies were solved by reexamination of the staining to achieve a consensus score. The number of positively stained cells in 10 representative microscopic fields was counted and the percentage of positive cells was calculated. The percentage scoring of immunoreactive tumor cells was as follows: 0, 0%; 1, <1%; 2, 1–10%; 3, 11–33%; 4, 34–67%; and 5, >67%. The staining intensity was visually scored and stratified as follows: 0, none; 1, weak; 2, moderate; and 3, strong. The final NNMT immunohistochemical score was presented as a composite (intensity + extent) (15). This overall score was then averaged with the number of 10 fields that were performed for each patient. An immunoreactivity (IR) score of 4 was defined as the cut-off for high NNMT IR to identify a potential correlation between NNMT expression in the malignant epithelium and the clinicopathological characteristics, as almost all benign foci exhibited IR scores of ≤3 for NNMT.

### Statistical analysis

Data are presented as the mean ± standard deviation. All statistical calculations were performed using SPSS version 11.0 software (SPSS, Inc., Chicago, IL, USA). Differences in the NNMT expression of three different types of prostate tissue were assessed using the Mann Whitney U test. Pearson’s χ^2^ test and Spearman’s correlation analysis were applied to investigate the association between NNMT expression and patient characteristics. Survival curves for patients exhibiting high and low NNMT expression were plotted using the Kaplan-Meier method, and statistical differences were compared using a log-rank test. Univariable and multivariable survival analyses were performed using the Cox regression analysis. P<0.05 was considered to indicate a statistically significant difference.

## Results

### Upregulated NNMT expression in HGPIN and PCa tissues

**Figure 1:**
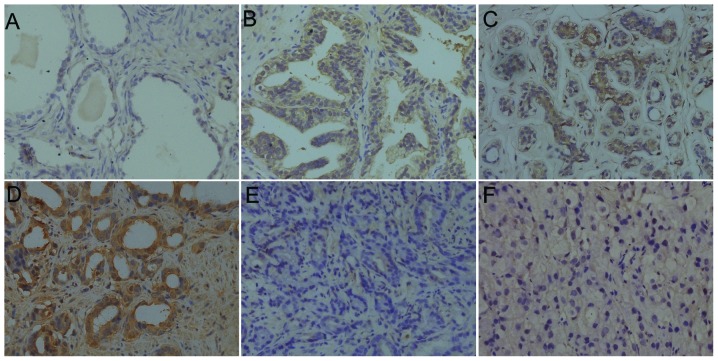
Pattern of NNMT expression revealed by immunohistochemical staining. (A) Negative NNMT expression was identified in BPH tissues, whereas (B) significant NNMT expression was identified in HGPIN tissues; (C–F) Different expression intensities in (C and D) well-, (E) moderately- and (F) poorly-differentiated PCa tissues (Gleason scores of 5, 6, 7 and 10, respectively). Magnification, ×200.

**Figure 2:**
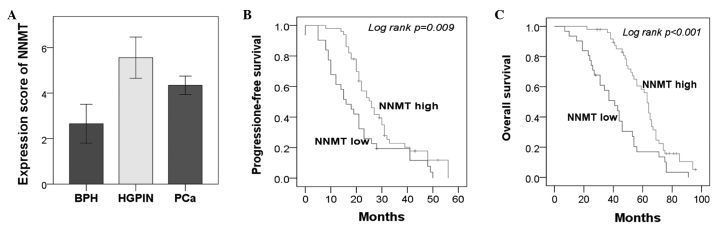
Statistical analysis. (A) Increased NNMT expression was identified in the HGPIN and PCa tissues compared with the BPH tissues (P<0.001 and P=0.001, respectively). (B and C) Kaplan-Meier analysis (B) progression-free survival and (C) overall survival curves according to NNMT expression in the advanced PCa cases (n=81). NNMT, Nicotinamide ***N***-methyltransferase; HGPIN, high-grade prostatic intraepithelial neoplasia; PCa, prostate cancer; BPH, benign prostate hyperlasia.

A total of 26 BPH, 18 HGPIN and 120 PCa tissues were evaluated using immunohistochemistry. While the majority of BPH cases (18/26; 69.2%) exhibited absent or extremely weak NNMT expression, NNMT was significantly upregulated in the HGPIN (15/18; 83.3%) and PCa (77/120; 64.2%) tissues, when using an IR score of 4 as a cutoff (Figs. 1 and 2A). The IR scores for the BPH, HGPIN and PCa tissues were 2.65±2.12, 5.56±1.82 and 4.34±2.26, respectively. The Mann Whitney U test revealed that NNMT expression in the HGPIN and PCa tissues was significantly higher than in the BPH tissues (P<0.001 and P=0.001, respectively). In carcinoma, NNMT was expressed at various intensities (Fig. 1C–F). The IR of NNMT was predominantly observed in the cytoplasm, however, it was also present in the nuclei of epithelial cells. No evident heterogeneity was identified in the staining pattern between duplicate tissue cores.

### Correlation between NNMT expression and clinicopathological variables in PCa

The association between NNMT expression and the clinicopathological factors of PCa, including patient age, serum PSA level, tumor stage, Gleason score, distant metastasis and lymph node metastasis, was investigated. Gleason score was found to negatively correlate with NNMT expression (P<0.001) (Table I). Spearman’s correlation analysis identified a correlation coefficient of −0.268 (P=0.003) between the IR and Gleason scores. However, no correlation was identified between other clinicopathological factors and NNMT expression.

### NNMT expression in advanced PCa correlates with prolonged PFS and OS time

Since the majority of specimens were obtained from patients with advanced PCa (81/120 cases; 67.5%), the prognosis of the patients with NNMT overexpression in this group was investigated. Kaplan-Meier analysis demonstrated that high NNMT expression was significantly associated with significantly prolonged PFS (P=0.009) and OS (P<0.001) times (Fig. 2B and C). In addition, the five-year OS rate was significantly higher in the NNMT high expression group compared with the NNMT low expression group [5/31 (16.1%) vs. 26/50 (52%); P=0.026]. Furthermore, these associations were confirmed by Cox univariate analysis [PFS: hazard ratio (HR), 0.54; 95% confidence interval (CI), 0.33–0.87; P=0.012; and OS: HR, 0.44; 95% CI, 0.27–0.71; P=0.001] and multivariate analysis, with adjustments for age, Gleason score, serum PSA level, distant metastasis, and lymph node metastasis (PFS: HR, 0.37; 95% CI, 0.20–0.67; P=0.001; and OS: HR, 0.29; 95% CI, 0.15–0.57; P<0.001) (Table II).

## Discussion

To the best of our knowledge, this study was the first to identify, using immunohistochemistry, that NNMT expression was significantly increased in HGPIN and PCa tissues compared with BPH tissues. Notably, NNMT expression was found to negatively correlate with the Gleason score. Furthermore, in advanced PCa, patients with high NNMT expression had significantly prolonged PFS and OS times compared with those exhibiting low NNMT expression.

NNMT catalyzes the transmethylation from *S*-adenosylmethionine to nicotinamide and to certain other azaheterocycles, playing a critical role in the biotransformation and detoxification of numerous xenobiotics. Usually, NNMT is highly expressed in the liver, whereas low expression has been identified in the kidneys, lungs, skeletal muscle, placenta, heart and brain. However, abnormal NNMT expression has been identified in several types of tumors, including glioblastoma (16), stomach adenocarcinoma (17), papillary thyroid cancers (18), colorectal cancer (12), hepatocellular carcinoma (19), lung cancer (11) and renal cancer (20), suggesting that NNMT may have a significant role in cancer. Among non-neoplastic disorders, the enhanced expression of NNMT has been detected in chronic obstructive pulmonary disease (21), atherosclerosis (22), and Parkinson’s disease (23).

In the present study, the expression of the NNMT enzyme was investigated in different prostatic tissues; those of BPH, HGPIN and PCa. While absent or weak in the majority of the BPH tissues, NNMT expression was significantly increased in the HGPIN and PCa tissues. HGPIN is the only premalignant precursor to PCa that has been identified. Similar genetic and molecular changes in HGPIN and PCa have been found (24). Notably, in the cancer tissues, this overexpression was more evident in the well-differentiated cases than in the undifferentiated cases. Furthermore, clinicopathological correlation analysis indicated that NNMT expression level was significantly associated with the Gleason score. Thus, these results suggested that the enzyme may be important in an initial step of the malignant conversion of PCa. These results were consistent with previous studies on oral squamous cell carcinoma (25).

The majority of advanced-stage PCa cases progress to castration-resistant prostate cancers. The treatment of advanced PCa remains a significant challenge. Establishing effective prognostic biomarkers may provide more information for the treatment of advanced PCa. The present study revealed that patients with tumors expressing high levels of NNMT exhibited a significantly prolonged PFS and OS times, which was also confirmed by multivariate analysis following adjustments for conventional prognostic factors. Therefore, aggressive treatment may be useful for advanced PCa patients that express low levels of NNMT. Considering the observation that NNMT expression is increased in HGPIN and well-differentiated PCa compared with BPH, we hypothesize that NNMT may exhibit different roles in the early stages of prostate cancer and during its progression toward metastatic or castrate-resistant states. Thus, we hypothesize that the underlying mechanisms for NNMT involvement in primary tumors may be distinct from metastatic tumors, and may function via different signaling pathways.

Due to its catalytic activity, NNMT may be involved in the regulation of intracellular levels of nicotinamide, 1-methylnicotinamide and *S*-adenosyl methionine. Nicotinamide is involved in the production of the coenzymes NAD(H) and NADP(H), which are essential for cellular functions. Several enzymes, which use NAD as substrate, including ADP-ribosyltransferases, CD38 and sirtuins, contain a nicotinamide-product site and may be inhibited by nicotinamide (26). In PCa cells, Sirtuin type 1 inhibition by nicotinamide has been shown to result in the significant inhibition of growth and viability, while exhibiting no effect on normal prostate epithelial cells (27). As a result of this inhibition, the salvage and/or elimination of nicotinamide is crucial for NAD metabolism, and thus, the NNMT enzyme may exhibit a crucial role in the regulation of these cellular events. NNMT activity may also be significant for the regulation of biological processes associated with 1-methylnicotinamide. In a previous study, overexpressing NNMT in SH-SY5Y neuroblastoma cells or incubating the cells with 1-methylnicotinamide significantly decreased the cell death rate, which was found to correlate with increased intracellular ATP content, ATP/ADP ratio and complex I activity, and reduced the degradation of complex I inhibitors (28), thus suggesting that 1-methylnicotinamide may mediate the cellular effects of NNMT. Recently, Ulanovskaya *et al* (29) showed that in cancer cells with high NNMT expression, NNMT impairs the methylation potential by consuming methyl units from *S*-adenosylmethionine, in order to generate the stable metabolic product, 1-methylnicotinamide. Consequently, NNMT-expressing cancer cells exhibit an altered epigenetic state, including hypomethylated histones and additional cancer-associated proteins combined with the increased expression of protumorigenic gene products. Therefore, inhibition of NNMT is considered to enhance the methylation potential and thereby may exhibit therapeutic benefits in cancers associated with insufficient histone methylation.

Based on previous studies, it appears that, via abnormal enzyme expression, the tumor cells regulate cellular processes associated with nicotinamide or 1-methylnicotinamide concentration or control transmethylation reactions using *S*-adenosylmethionine (25). In the present study on advanced PCa, improved PFS and OS times were revealed for patients bearing tumors with higher NNMT expression levels compared with patients with tumors with lower NNMT expression. Thus, the identification of the NNMT expression status is likely to aid the prediction of the prognosis for each patient.

In conclusion, the present study suggests that, although a preliminary result, NNMT expression has significant potential for the development of PCa treatment. In addition, high NNMT expression levels are considered to result in increased PFS and OS times for patients with advanced PCa. Further studies are required to assess its promising role as a novel therapeutic drug target or prognosis biomarker for PCa.

## Figures and Tables

**Table I:** Association between NNMT expression and the clinicopathological parameters of PCa.

		NNMT expression, n (%)		
			
Characteristics	Patients, n	High	Low	P-value
Age, years				0.219
<69	61	40 (65.6)	21 (34.4)	
≥69	59	33 (55.9)	26 (44.1)	
PSA, ng/ml				0.808
≤10	21	14 (66.7)	7 (33.3)	
>10	99	59 (59.6)	40 (40.4)	
Distant metastasis (pM1)				0.452
Yes	56	32 (57.1)	24 (42.9)	
No	64	41 (64.1)	23 (35.9)	
Lymph metastasis (pN1)				0.385
Yes	28	19 (67.9)	9 (32.1)	
No	92	54 (58.7)	38 (41.3)	
pT-status				0.442
pT1/2	25	17 (68.0)	8 (32.0)	
pT3/4	95	56 (58.9)	39 (41.1)	
Gleason score				<0.001^a^
5–6	31	28 (90.3)	3 (9.7)	
7	21	15 (71.4)	6 (28.6)	
8–10	68	30 (44.1)	38 (55.9)	

a P<0.05.

NNMT, Nicotinamide ***N***-methyltransferase; PCa, prostate cancer; PSA, prostate-specific antigen.

**Table II:** Cox univariate and multivariate analysis of PFS and OS according to NNMT expression.

	Univariate (PFS)		Multivariate (PFS)		Univariate (OS)		Multivariate (OS)	
				
Variables	HR	P-value	HR	P-value	HR	P-value	HR	P-value
Age, years (<60 vs. ≥60)	1.27 (0.79–2.05)	0.322	1.21 (0.75–1.97)	0.435	1.50 (0.93–2.42)	0.099	1.81 (1.08–3.03)	0.024^a^
Gleason score (5–6 vs. 7 or 8–10)	1.54 (1.07–2.22)	0.021^a^	1.59 (1.05–2.41)	0.028^a^	1.71 (1.17–2.49)	0.005^a^	1.78 (1.07–2.62)	0.023^a^
PSA, ng/ml (≤10 vs. >10)	2.70 (1.32–5.53)	0.007^a^	5.91 (2.52–13.85)	<0.001^a^	2.20 (1.12–4.29)	0.021^a^	6.09 (2.66–18.96)	<0.001^a^
Distant metastasis (yes vs. no)	2.24 (1.30–3.86)	0.004^a^	2.53 (1.44–4.45)	0.001^a^	2.01 (1.20–3.35)	0.008^a^	2.29 (1.34–3.90)	0.002^a^
Lymph node metastasis (yes vs. no)	1.82 (1.08–3.06)	0.024^a^	2.03 (1.19–3.45)	0.010^a^	2.19 (1.29–3.72)	0.004^a^	2.59 (1.51–4.47)	0.001^a^
NNMT expression (high vs. low)	0.54 (0.33–0.87)	0.012^a^	0.37 (0.20–0.67)	0.001^a^	0.44 (0.27–0.71)	0.001^a^	0.29 (0.15–0.57)	<0.001^a^

a P<0.05.

PSA, prostate-specific antigen; PFS, progression-free survival; OS, overall survival; HR, hazard ratio; 95% CI, 95% confidence interval; NNMT, Nicotinamide *N*-methyltransferase.
